# A miRNA-based epigenetic molecular clock for biological skin-age prediction

**DOI:** 10.1007/s00403-024-03129-3

**Published:** 2024-06-01

**Authors:** Jose Vicente Roig-Genoves, José Luis García-Giménez, Salvador Mena-Molla

**Affiliations:** 1 Rara Avis Biotec S.L, Parc Científic de la Universitat de Valencia, Paterna, 46980 Spain; 2 Consortium Center for Biomedical Network Research on Rare Diseases (CIBERER), Institute of Health Carlos III, Valencia, 46010 Spain; 3 INCLIVA Health Research Institute, INCLIVA, Valencia, 46010 Spain; 4 EpiDisease S.L (Spin-off from the CIBER-ISCIII), Parc Científic de la Universitat de Valencia, Paterna, 46980 Spain; 5 Department of Physiology, Faculty of Pharmacy, University of Valencia, Burjassot, 46100 Spain

**Keywords:** Aging, Skin, Machine learning, Prediction, ElasticNet, Suppor Vector classifier

## Abstract

Skin aging is one of the visible characteristics of the aging process in humans. In recent years, different biological clocks have been generated based on protein or epigenetic markers, but few have focused on biological age in the skin. Arrest the aging process or even being able to restore an organism from an older to a younger stage is one of the main challenges in the last 20 years in biomedical research. We have implemented several machine learning models, including regression and classification algorithms, in order to create an epigenetic molecular clock based on miRNA expression profiles of healthy subjects to predict biological age-related to skin. Our best models are capable of classifying skin samples according to age groups (18–28; 29–39; 40–50; 51–60 or 61–83 years old) with an accuracy of 80% or predict age with a mean absolute error of 10.89 years using the expression levels of 1856 unique miRNAs. Our results suggest that this kind of epigenetic clocks arises as a promising tool with several applications in the pharmaco-cosmetic industry.

**Supplementary Information:**

The online version contains supplementary material available at 10.1007/s00403-024-03129-3.

## Introduction

Aging is a complex multifactorial biological phenomenon characterized by a progressive functional, metabolic and physiological decline at the molecular, cellular, tissue, and organ level produced over time with advancing chronological age [1]. These functional changes take place at the molecular level, setting the basis of biological aging. Biological aging has been molecularly characterized according to 9 biological hallmarks of aging comprising genomic instability, telomere attrition, epigenetic alterations, loss of proteostasis, deregulated nutrient-sensing, mitochondrial dysfunction, cellular senescence, stem cell exhaustion, and altered intercellular communication, which are interconnected among them [2]. Aging can occur at different rates in different individuals, and even between different tissues of the same individual.

Skin is the most extensive organ of our body, and it provides us a protective barrier from the environment, maintaining general homeostasis, and it also controls immune activity and sensory perception. The increased life expectancy, in addition to the continuous skin exposition to harmful environmental factors such as solar ultraviolet irradiation (UV), can lead to skin damage inducing loss of barrier function, with dryness, cell death with a lower rate of renewal, thinning and loss of elasticity, leading to skin fragility and wrinkles, and aging-related skin diseases such as psoriasis, rosacea, atopic dermatitis, melanoma, among others [3]. These skin disorders can affect self-esteem and quality of life, including social life, with important implications on the psychological health of the affected population [4], which has been accentuated in in the last decades given the increase in life expectancy.

To prevent or to avoid these skin damages, we need to either stop the aging process or be able to revert an organism from an older to a younger stage (the long-awaited rejuvenation), one of the most important challenges for both pharma and dermo-cosmetic industry.

However, in order to achieve an effective anti-aging treatment, it is necessary to understand what is occurring at the molecular level in the skin of each individual before the phenotype is even affected.

It has been demonstrated that epigenetics (such as changes in histone modifications, organization of chromatin, DNA methylation and non-coding RNAs) has a crucial role in aging. Epigenetics mechanisms connect genotype and phenotype, can undergo specific and dynamic alterations in specific tissues and, additionally, epigenetic mechanisms can be modulated by changes in lifestyle (such as exercise or nutrition) or pharmacologically, improving both longevity and health span [2, 5–7] making them an excellent target for the study of aging.

Different clocks have been developed in order to assess aging or even risk of death [8]. Among them, the most common are those based on DNA methylation, such as the Hannum or Horvath’s clock [9]. However, in the upper layers of the epidermis, prior to the synthesis of the enucleate cornified layer which confers the majority of epidermal barrier function [10], the nucleus of keratinocytes are degraded. This involves, that upper layers of keratinocytes do not have nuclear DNA, probably, does not faithfully represent the aging state of the skin. In fact, in some common pathologies of skin associated with accelerated aging, such as psoriasis, methylation-based clocks have not shown good prediction [11].

Micro RNAs (miRNAs) are small non-coding RNAs that regulate the gene expression by repressing the translation and/or degradation of their messenger RNA (mRNA) target. The changes in both miRNAs and their target mRNAs have been evaluated in human epidermal keratinocyte and fibroblasts senescence during skin aging [12].

In this context, we have developed a miRNA-based epigenetic molecular clock to predict biological age and characterize the skin-aging stage through the characterization of miRNA signature of healthy people using high-throughput sequencing data of miRNA, a type of small non-coding RNA molecules, obtained from the Gene Expression Omnibus (GEO) repository from the National Center for Biotechnology Information (NCBI) of the United States of America (U.S.). The applications of this epigenetic molecular clock based on miRNA will allow to identify key molecular mechanisms underlying skin aging and therefore to evaluate potential therapeutic approaches.

## Methods

### Data retrieval and processing

Datasets containing raw data from miRNA expression profiles from healthy skin biopsies with chronological age available and obtained throughout small RNA high-throughput sequencing (HTS) were selected from GEO (https://www.ncbi.nlm.nih.gov/geo/).

The sequencing data pre-processing steps applied to data were the following. For the first quality control (QC) of raw data, FastQC software v0.11.9 [13] was used, obtaining one quality assessment report for each sample. Afterward, data were preprocessed using Cutadapt software v1.18 [14] with the following parameters: -m 16 to discard processed reads that are shorter than 16 base pairs (bp), -q 20 for base quality filtering (Phred quality score), and adapter trimming. Only reads with a minimum length of 16 bp were selected for further analyses. Subsequently, second quality control was applied to all samples using the same tool as previously. After preprocessing, reads were aligned to the human reference genome (hg38) using Subread software v2.0.1 [15] applying the specific recommendations for miRNA-seq (see Subread manual). Then, reads were annotated using a small RNA database human miRBase 21 [16] via *R software* (v4.0) and RSubread package v2.2.6. Feature selection was based on selecting only those miRNAs which present at least a minimum of 10 read counts across 5% of all samples. Data were normalized using variance stabilizing normalization (VST) method using DaMiRseq v2.0.0 *R* packages. Finally, batch effect correction and surrogate variables adjust were applied to remove unwanted technical issues of samples, using DaMiRseq package, which internally uses *removeBatchEffect* function from limma v3.13 package.

### Epigenetic clock development

We have adapted 8 machine learning methods, including regression and classification algorithms, for biological age prediction based on miRNA expression profile: Regression ((Elastic Net (EN), AdaBoost (AB), Support Vector Regression (SVR) and Lasso), and classification (Random Forest Classifier (RFC), Gradient Boosting Classifier (GBC), Support Vector Classification (SVC) and k-Nearest Neighbors (k-NN)). These algorithms have been widely used to solve various classification and prediction problems in biology [17–20].

The data were structured according to each machine learning approach. For regression tasks, the data matrix was structured on one row per sample and one column per miRNA. For each sample, the chronological age was included in the last column. On the other hand, for classification tasks, samples were restructured into 5 different decade groups (18–28; 29–39; 40–50; 51–60 and 61–83), that allows groups with a similar size and covers the age range of the samples available in the datasets. All models were implemented by the sci-kit-learn library using their implementation in scikit-learn v0.24.1 [21] in Python v3.7.6.

Machine learning models were trained on normalized, adjusted for unwanted batch effects and scaled expression profile values of selected miRNAs expressed across samples. The dataset, was split, at a ratio of 80/20, into training and testing sets.

For model hyperparameter optimization, we used a grid search of the hyperparameter space with a nested cross-validation approach in our specific dataset for each model.

The model performance evaluation was done separately using 10-fold cross-validation for regression models and 5-fold cross-validation for classification models. The metrics used to evaluate the accuracy of regression algorithms were: Mean absolute error (MAE), which is the average of the absolute differences between predictions and actual values; Coefficient of determination (*R*^*2*^); and Root Mean Squared Error (RMSE).

For classification algorithms, the metrics used to evaluate their accuracy were: Confusion matrix, which is a presentation of the accuracy of the model with multiclass classification problems; Accuracy, which is the classifier’s ability to correctly predict the class of each sample; f1 score, which is the weighted average between precision and recall; Recall, which is the number of correct predictions divided by the total number of elements present in that class.

All models were trained on the same training dataset. To predict the age of the testing dataset, the individual’s miRNA expression profile levels are given as input for each model.

In order to avoid the effect of imbalanced dataset, one of the problems in biological classification methods [22–24], we applied the Synthetic Minority Oversampling Technique (SMOTE) [25] using imblearn library v0.5.0 in Python. Briefly, SMOTE is a method of synthetic over-sample, it works through selecting samples from the minority class that is close in the feature space, then, it finds its 5 nearest neighbours of that samples and a new sample is synthetically created at a randomly selected point between the selected samples in the feature space.

## Results

### Epigenetic clock miRNA-based database development

In the GEO repository, we find more than 6400 datasets of which more than 430 correspond to miRNA data on humans. Data were selected according to following eligibility criteria: small RNA profiling data which was obtained by high throughput sequencing from skin biopsies on human healthy subjects with available chronological age. This selection process resulted in 72 healthy samples with publicly miRNA data and available information about chronological age.

Initial screening and quality filtering according to “Methods” section were applied, resulting in 64 samples of 18 to 83 years old with an average of 45.20 (± 16.94) that fit with the eligibility and sequencing data quality criteria. The four datasets used were: GSE31037 [26], GSE72193 [27], GSE84193 [28], GSE142582 [29] (Suppl. Table 1).

**Fig. 1:**
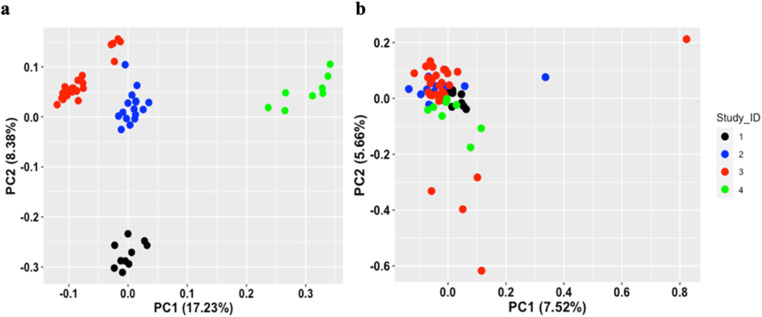
Principal Component Analysis (PCA) of miRNAs database. **a**) PCA depicting the distribution of samples before batch effect correction and adjusting for surrogate variables. **b**) PCA depicting the distribution of samples after batch effect correction. Study_ID 1: GSE31037 (Joyce et al. 2011).Study_ID 2: GSE72193 (Gulati et al. 2016). Study_ID 3: GSE84193 (Chitsazzadeh et al. 2016). Study_ID 4: GSE142582 (Yu et al. 2020b)

Metadata was analysed according pipeline described in “Methods” section and Suppl. Figure 1. After normalization (Suppl. Figure 2), Principle component analysis (PCA) plots illustrated sample distribution and the need for a batch effect correction (Fig. 1).

Raw miRNA annotation resulted in 2652 unique miRNAs that were present in at least in one of the samples. Notice that, to date, nearly 2700 human mature miRNAs have been identified [30]. These miRNAs were filtered by expression and consistency expression across samples, resulting in 1856 unique miRNAs used in next steps (Suppl. Table 2).

### Machine learning algorithms to predict biological age from miRNA expression profile

Two different approaches were developed in order to predict chronological age from a skin-related miRNA expression profile, using miRNAs expression values, regression algorithms to predict exactly the biological age, or classification algorithms to classify each individual in different age groups.

We have used 6 different regression algorithms, ElasticNet, Lasso, AdaBoost Regressor and 3 different kernel function in SVR model, the Radial basis (RBF), lineal, and polynomial kernel functions (Table 1). Moreover 4 different multiclass classification algorithms have been performed, Random Forest Classifier (RFC), Suppor Vector Classifier (SVC), k-nearest neighbors (kNN), and Gradient Boosting Classifier (GBC) (Table 2).

**Table 1:** Model evaluation metrics for regression models

Regression model		Evaluation metrics		
		MAE (years)	*R* ^2^	RMSE
*ElasticNet*		10.89 ± 4.09	0.53 ± 0.39	14.17 ± 5.30
*Lasso*		11.57 ± 4.11	0.45 ± 0.37	14.69 ± 5.32
*AdaBoost Regressor*		11.10 ± 2.90	0.31 ± 0.17	13.28 ± 3.29
*Support Vector Regressor*	*RBF*	11.19 ± 2.77	0.33 ± 0.20	13.28 ± 2.85
	*Lineal*	19.47 ± 4.59	0.24 ± 0.17	25.61 ± 1.72
	*Polynomial*	13.18 ± 4.10	0.22 ± 0.27	16.27 ± 4.56

RBF: Radial basis kernel function. MAE: Mean absolute error in years, mean ± standard deviation). R^2^: Coefficient of determination, mean ± standard deviation. RMSE: root-mean-square-error in years, mean ± standard deviation. Optimal hyperparameters for EslasticNet: Alpha of 10, and L1 ratio of 0.01. Optimal hyperparameters for Lasso: Alpha of 0.9. Optimal hyperparameters for AdaBosst Regressor: Number of estimators of 500, and Learning rate of 1. Optimal hyperparameters for Support Vector Regressor (SVR)-RBF: Radial basis kernel, C of 1000 and gamma of 0.0001. Optimal hyperparameters for SVR-Lineal: Lineal kernel, C of 0.1, and gamma of 1. Optimal hyperparameters for SVR-Polynomial: Polynomial kernel, C of 1 and gamma 0.001

**Table 2:** Model evaluation metrics for classification models

Classification model		Evaluation metrics						
		**Accuracy**		**f1**		**Recall**		
			**Micro**	**Macro**	**Weighted**	**Micro**	**Macro**	**Weighted**
*Epigenetic clock dataset*	*RFC*	38.3% ± 12.4%	0.38 ± 0.13	0.25 ± 0.09	0.30 ± 0.12	0.38 ± 0.11	0.29 ± 0.09	0.38 ± 0.12
	*SVC*	38.7% ± 12.8%	0.39 ± 0.13	0.24 ± 0.14	0.29 ± 0.13	0.39 ± 0.13	0.29 ± 0.13	0.39 ± 0.13
	*kNN*	30.8% ± 11.8%	0.31 ± 0.12	0.17 ± 0.10	0.23 ± 0.11	0.31 ± 0.12	0.22 ± 0.10	0.17 ± 0.10
	*GBC*	39.3% ± 11.2%	0.38 ± 0.12	0.26 ± 0.12	0.30 ± 0.12	0.38 ± 0.12	0.26 ± 0.12	0.26 ± 0.12
*Epigenetic clock dataset + SMOTE*	*RFC*	76.8% ± 11.1%	0.76 ± 0.10	0.77 ± 0.10	0.76 ± 0.10	0.77 ± 0.11	0.75 ± 0.11	0.77 ± 0.11
	*SVC*	80.8% ± 8.7%	0.81 ± 0.09	0.81 ± 0.08	0.81 ± 0.08	0.81 ± 0.09	0.29 ± 0.13	0.81 ± 0.09
	*kNN*	64.2% ± 8.1%	0.64 ± 0.08	0.58 ± 0.08	0.58 ± 0.08	0.64 ± 0.08	0.64 ± 0.08	0.64 ± 0.08
	*GBC*	77% ± 9.5%	0.79 ± 0.08	0.77 ± 0.09	0.77 ± 0.10	0.31 ± 0.11	0.77 ± 0.09	0.77 ± 0.10

RFC: Random Forest Classifier. SVC: Suppor Vector Classifier. kNN: k-nearest neighbors. GBC: Gradient Boosting Classifier. Optimal hyperparameters for RFC: Max_features as square root, without maximum depth of the tree, and 1000 estimators. Optimal hyperparameters for SVC: Radial basis kernel, C of 50 and scale as gamma. Optimal hyperparameters for kNN: Manhattan as metric, number of neighbours as 13, distance as weight. Optimal hyperparameters for GBC: Learning rate of 0.1, maximum depth of 3, number of estimators 1000, and subsample of 0.5. SMOTE: Synthetic Minority Oversampling Technique. Mean ± standard deviation

#### Regression models

**Fig. 2:**
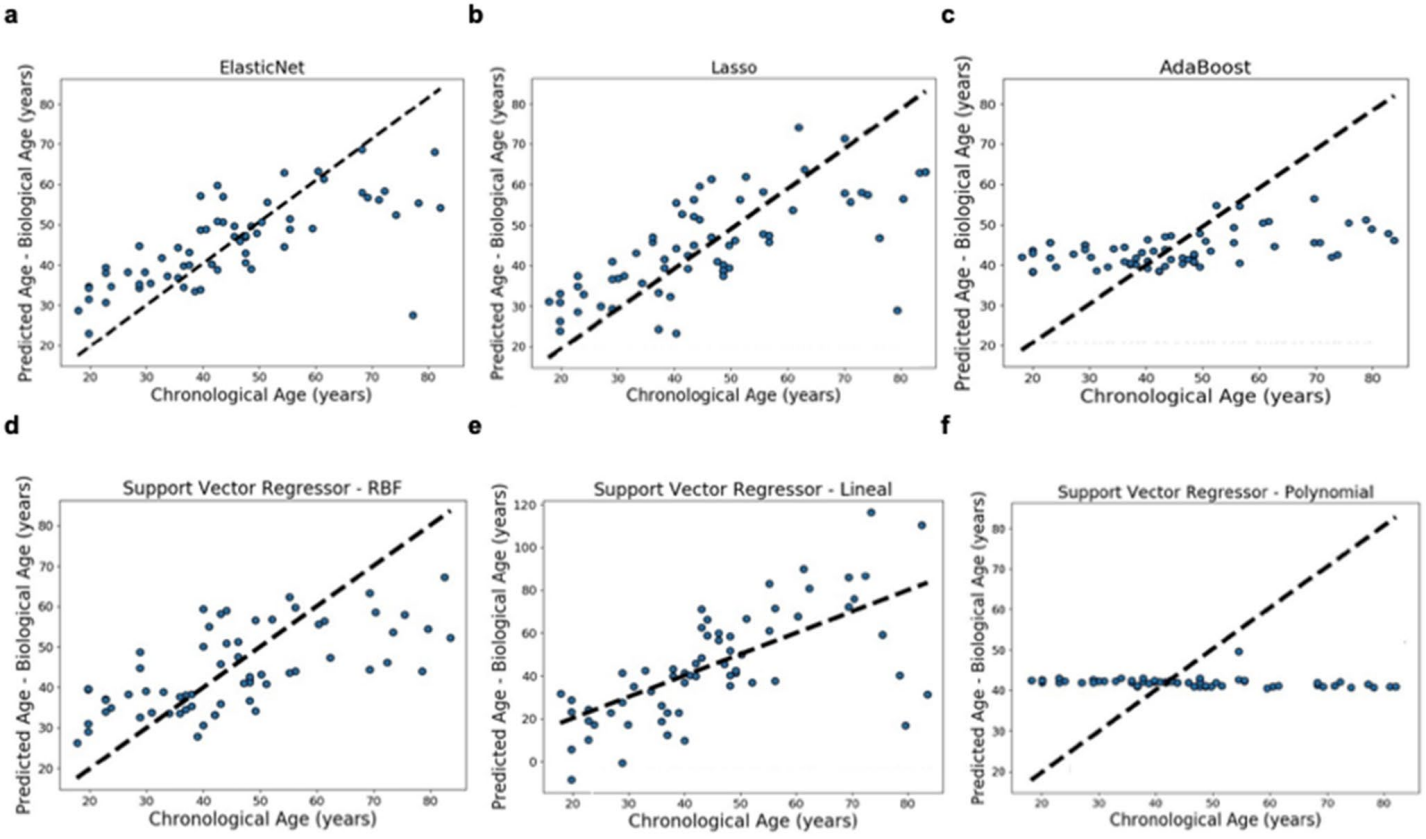
Biological age prediction from miRNA expression by regression analysis. Results of the biological age prediction, using regression models, computed by 10 cross-validation. The x-axis shows the chronological age in years. The y-axis show the biological age in years (predicted age) computed for each regression model **a**) ElasticNet, **b**) AdaBoost regressor, **c**) Lasso, **d**) Support Vector Regressor with radial basis kernel function (SVR-RBF), (e) SVR with lineal kernel function, (f) SVR with polynomial kernel function. Every blue dot depicts one prediction for one subject of the testing set during the cross-validation. The dotted line shows the perfect linear correlation

Each regression model assigns a predicted age according to its miRNA expression profile. The plots in Fig. 2 show the boundaries of each regression model prediction of the chronological age for testing datasets, using an optimal hyperparameters setting for each model and their evaluation after a grid search with a nested 10 cross-validation approach. The models were built on 80% of the total dataset and the remaining 20% were used for testing, in each fold of the cross-validation.

ElasticNet and Lasso (Fig. 2a and b) models seemed to be the best algorithms for age prediction with the current dataset. In the case of ElasticNet with an R^2^ of 0.53, a MAE of 10.89 years, and a RMSE of 14.17 years. And for the Lasso model, with an R^2^ of 0.45, MAE of 11.57, and RMSE of 14.69. Interestingly, these models predicted with high accuracy individuals from 18 to 70 years old, however, with older individuals, variance in the results started to grow up.

AdaBoost model (Fig. 2c) showed a prediction bias within the interval of 30–55 years old. However, the MAE value (11.10) was similar to those obtained ElasticNet and Lasso models, probably due to sample overrepresentation of this range of ages.

The performance of the SVR-RBF (Fig. 2d) was similar to the best models, with and reduced R^2^ of 0.33, MAE of 11.19, and RMSE of 13.28, nevertheless it presented an early age prediction deviation with older samples around 55 years.

SVR models with linear and polynomial functions (Fig. 2e and f) showed the worst performance and were discarded for validation analysis.

#### Multiclass classification models

A different data structure was used to work with multiclass classification models. Samples were associated into 5 different age groups obtaining a 5-class classification problem: 18–28, 29–39, 40–50, 51–60, 61–83 (Table 3). Although this distribution is the most balanced possible distribution of samples, it results in an unbalanced dataset, being group 3 the most represented class with 21 samples between 40 and 50 years old, and group 4 (51–60 years old) the less represented one with 7 samples.

**Table 3:** Sample distribution for machine learning classification multiclass classification tasks

Sample groups for classification machine learning algorithms					
Group	Group 1	Group 2	Group 3	Group 4	Group 5
Age range	18-28	29-39	40-50	51-60	61-83
Number of samples	10	14	21	7	12

In that context, we have built 4 different classification algorithms for this 5-class classification problem. Optimal model hyperparameters for each model obtained after a grid search with a 5-cross validation, as well as the evaluation of the models using the dataset, are depicted in Table 2.

For model evaluation, precision, recall, and f1 metrics were calculated. Moreover, we used micro, macro, and weighted averaging metrics. Micro averaging calculates metrics globally by counting the total true positives, false negatives, and false positives. Macro averaging calculates metrics for each class, and therefore, it does not take into account class imbalance. Weighted averaging is identical to macro but including the occurrence ratio for each class.

The precision of classification models resulted similar, being GBC the best model with 39.3% of accuracy. SVC and RFC also presented an accuracy close to the GBC model with 38.7% and 38.3%, respectively. k-NN showed the worst accuracy value of 30.8%.

Table 2 depicts the evaluation of the identical models with SMOTE approach applied to balance the dataset. After SMOTE, new 41 synthetic samples were generated to balance the classes obtaining 21 samples in each class with a total of 105 samples.

The model performance has considerably increased in all cases, SVC resulted in an accuracy of 80.8% increasing 42.1% concerning unbalanced results. Similarly, RFC increased 38.5 points achieving an accuracy of 76.8%.

**Fig. 3:**
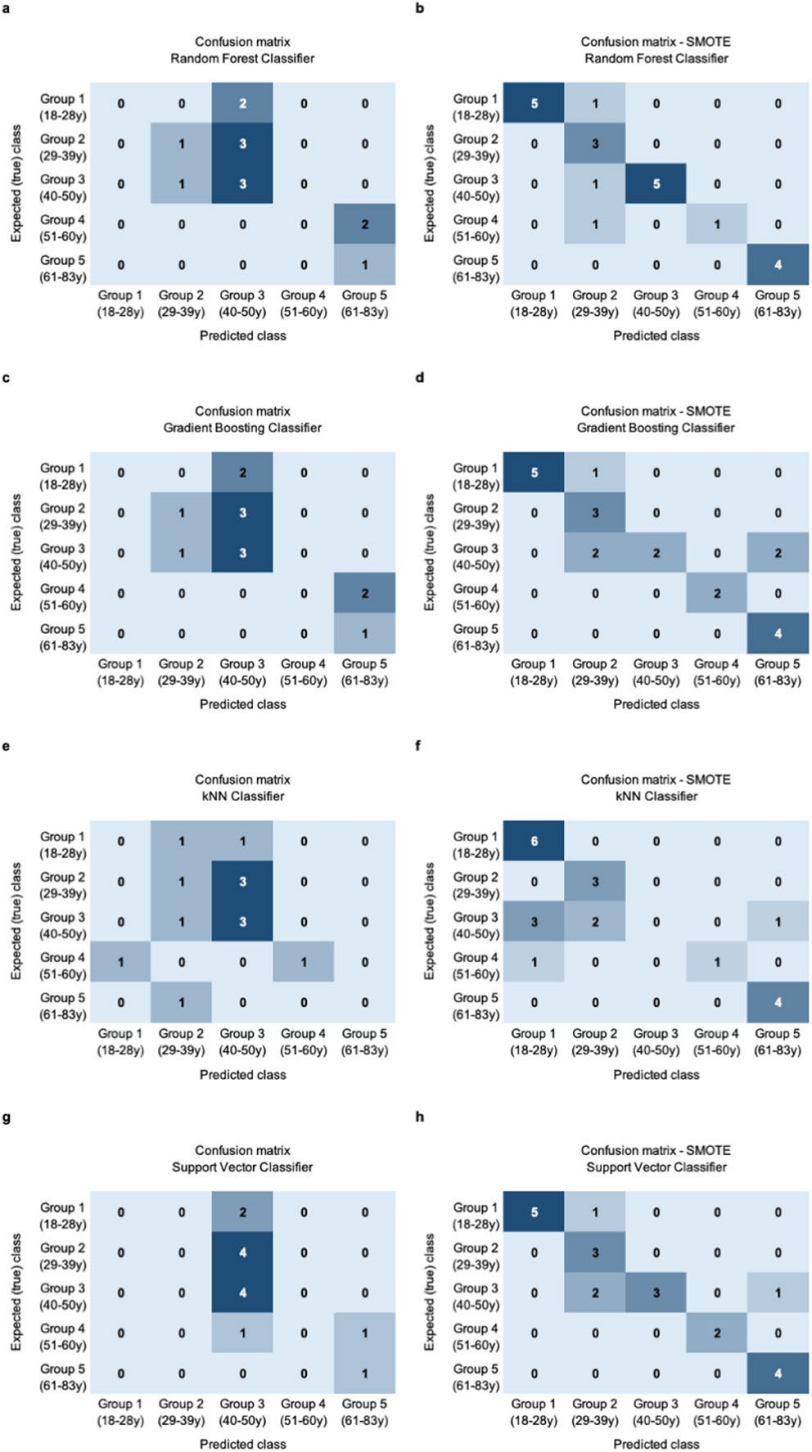
Confusion Matrix of classification models for biological age group. **a, c, e, g**. Confusion matrix for multiclass classification models (Random Forest, Gradient Boosting, kNN, and Support Vector Classifier) after age group prediction of a common training set (13 samples). The x-axis shows the true label (real age group) for a given sample. The y-axis shows the predicted label (predicted age group) for a given sample according to age groups. Group 1: 18–28 years old. Group 2: 29–39 years old. Group 3: 40–50 years old. Group 4: 51–60 years old. Group 5: 61–83 years old. **b, d, f, h**. Confusion matrix for multiclass classification models (Random Forest, Gradient Boosting, kNN, and Support Vector Classifier) with SMOTE application after age group prediction for a common training set (21 samples). The x-axis shows the true label (real age group) for a given sample. The y-axis shows the predicted label (predicted age group) for a given sample. The optimum confusion matrix should show a perfect diagonal line

In the case of GBC and k-NN the application of SMOTE also increased moderately the performance of the models, GBC presents 77% accuracy (39.3% with the original dataset), and k-NN improved the accuracy in 33.4 points until 64.2% of accuracy (Fig. 3).

## Discussion

The present work described arises an epigenetic clock based on skin-related miRNA expression levels profile to predict biological age. Our epigenetic clock (ElasticNet, MAE 10.89 years, *N* = 64 samples) can predict biological age with similar accuracy, in terms of MAE, to comparable biological clocks based on mRNA in fibroblasts (MAE 7.7 years) [19], blood transcriptome (MAE 7.8 years) [31], DNA methylation from skin biopsy samples (MAE 3.9 years for epidermis or 4.1 years for dermis) [32], or even protein biological clock (RMSE 11.3 years) [33]. Although some clocks based on DNA methylation present better results, we must highlight that: (1) Epidermal or dermis samples could present better accuracy than whole skin biopsy [34]; (2) In addition, upper layers of keratinocytes do not have nuclear DNA so the epigenetic clock based on the use of DNA methylation is not a good option for skin’s clock [11, 35]; (3) In some common pathologies of skin associated with accelerated aging, such as psoriasis, methylation-based clocks have not shown good prediction [11, 35]. Therefore, despite biological clocks already existing, it is necessary to continue researching in skin-aging biomarkers to improve biological skin-aging clock that faithfully and dynamically reflects the aging state of the skin and that allows the development of effective anti-aging solutions.

Our results demonstrated that ElasticNet algorithm obtain better accuracy than other machine learning algorithms as Lasso, AdaBoost or SVR-RBF models.

ElasticNet model trained with the current dataset (64 samples) presents good performance for biological age prediction for subjects aged between 18 and 70 years old. For subjects over 70, there is a less accurate correlation between chronological and biological age. This fact, probably, could be due to the minority representation of matched ages subjects in the dataset. However, the accumulation of epigenetic alteration in aging cells, specifically in skin cells that are continuously exposed to environmental factors, during the aging, could lead to this high variability in the miRNome composition for elderly subjects [19, 29].

The increased variance presented for older samples has been also described in similar studies and is extremely related to the lack of validated aging biomarkers [19, 29]. Our skin-aging performance prediction is promising since it has demonstrated that the application of machine learning algorithms to smallRNA-seq data can provide new insight into the epigenetic field to obtain a better understanding of the skin aging process and skin-related diseases as it is done with transcriptome [36]. However, like other authors, our current model cannot be used to evaluate age acceleration either, especially in those cases in which the results are within the error of predictive accuracy [5], due to the high MAE obtained.

Classification models will play a crucial role in this epigenetic clock approach. The results obtained with classification models show better predictions than regression models using the same dataset. Presumably, the unbalanced distribution of data between age groups, and the age range of each group (10 years) are responsible for this low accuracy since unbalance datasets directly affect the boundary of classification models to correctly classify samples in minority groups since models will be biased in favour of the majority class. For that reason, we used SMOTE approach [25], to balance our dataset.

This assumption has been demonstrated and confirmed with SMOTE approach, where 41 new synthetic samples were generated to balance the dataset and therefore, model accuracy improves significantly until 80.8% with SVC (Fig. 3; Table 3).

The development of skin-specific miRNA databases with a greater number of samples and balanced in the different age ranges would allow the application of classification models. Hence, the confidence of the prediction of the biological age and the classification of the subjects by the classification models would increase, as well as it would allow us to perform even more personalized differential expression analysis (DEA) identifying specific pathways and biomarkers involved in the skin-aging process and skin-related diseases.

The characterization of the gap between chronological and biological age, as well as, the molecular pathways responsible for this discrepancy in comparison with the suitable miRNA profile for each age will allow creating individualized advice to prevent skin aging and recover a healthy and younger skin-related miRNome.

Improving the comprehension of the process responsible of aging is one of the current challenges in aging and biomedical research. In this context, our pipeline brings a new tool to face up this problem, offering the first tool for accurately predicting an individual’s biological skin-age, and for analyzing the main pathways involved in the skin-related aging process in humans in order to improve interventions for restoring epidermal homeostasis and ameliorating disease burden in severe skin conditions [37].

## Conclusion

We have shown that it is possible to generate a biological clock using miRNA profiles, obtaining correlations similar to those achieved with biological clocks based on mRNA or DNA methylation. However, the greater stability of miRNAs compared to mRNAs suggests that biological clocks based on miRNAs could provide more robust results. On the other hand, unlike DNA, miRNAs are present in the upper layers of the skin, so miRNA analysis could be used to assess skin status. The approximation of that pipeline offers a new tool for individualized skin health care. Our pipeline facilitates the development of new monitoring and prognostic tools for skin-related diseases, and provides to the pharmaceutical industry a new platform to evaluate potential therapies and to identify optimum individualized treatments for each subject.

### Electronic supplementary material

Below is the link to the electronic supplementary material.

Supplementary Material 1

Supplementary Material 2

Supplementary Material 3

## Data Availability

Our study establishes an epigenetic molecular clock based on microRNA (miRNA) signature for biological skin-age prediction. The integration of this epigenetic clock through regression model and classification model uses a bioinformatic pipeline to smallRNA-seq data preprocessing and analysis, allow us to characterize and identify key molecular mechanisms underlying skin aging as well as it arises as new platform for molecularly test potential antiaging compounds to evaluate potential therapeutic approaches. We used publicly available datasets. The details can be found in Supplementary Table S1.
