# Supplementary material for: A miRNA-based epigenetic molecular clock for biological skin-age prediction
Source: Arch Dermatol Res. 2024 Jun 1;316(6):326. doi: 10.1007/s00403-024-03129-3 (PMC11144124; doi:10.1007/s00403-024-03129-3)
Supplement: Supplementary file 1 — Supplementary Material 1 [file 403_2024_3129_MOESM1_ESM.docx]

## Supplementary material


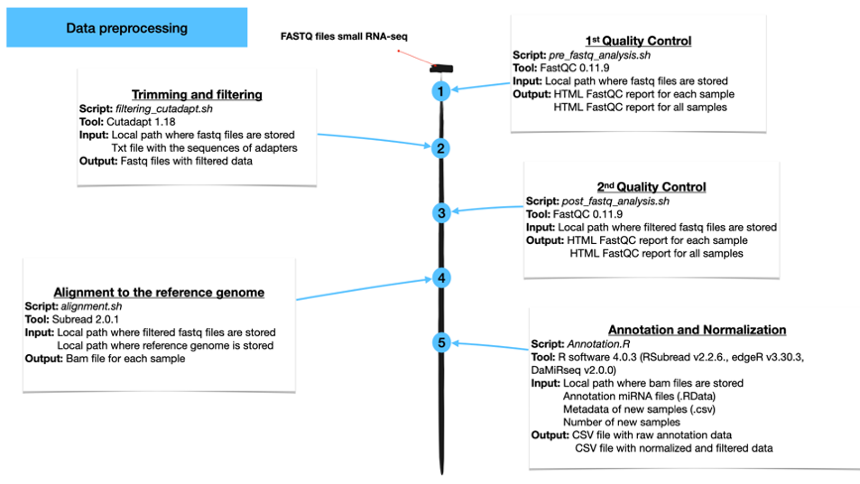


**Supplementary Figure 1:** Data processing workflow for small RNA-seq dataset development. All requirements, scripts and outputs are depicted for each step


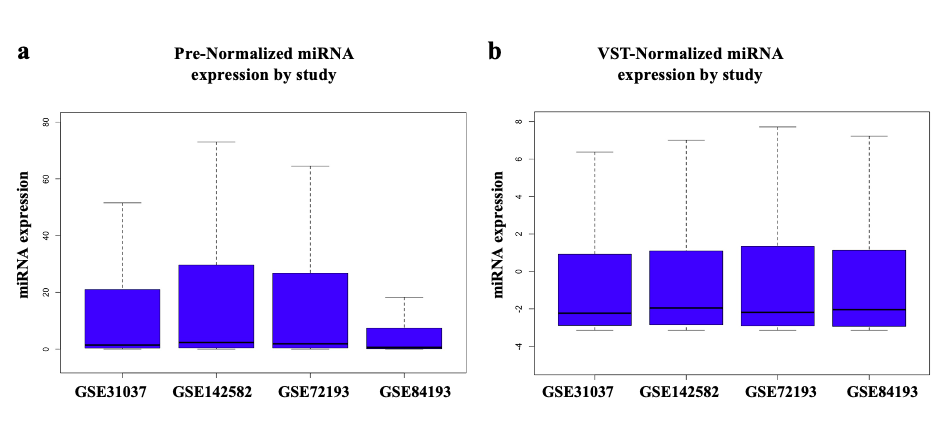


**Supplementary Figure 2:** Comparison of raw data (a) and VST normalized data (b) according to Section 2.1.

***Supplementary Table 1*:** 64 samples that fit with the eligibility and sequencing data quality criteria.

***Supplementary Table 2*:** 1856 unique miRNAs used for epigenetic biological clock generation.
